# BtsCI and BseGI display sequence preference in the nucleotides flanking the recognition sequence

**DOI:** 10.1371/journal.pone.0202057

**Published:** 2018-08-17

**Authors:** João Rosa, Esther Fernandez-Gonzalez, Cosimo Ducani, Björn Högberg

**Affiliations:** Department of Medical Biochemistry and Biophysics, Karolinska Institutet, Stockholm, Sweden; University of Helsinki, FINLAND

## Abstract

Restriction enzymes are the bread and butter of Molecular Biology. Nonetheless, how restriction enzymes recognize and cleave their target is not always clear. When developing a method for the enzymatic production of oligonucleotides, we noticed that type II endonucleases BtsCI and BseGI, which recognize the sequence GGATGNN^, perform incomplete digestions of DNA hairpins, with the top strand nick not always occurring correctly. We tested the cutting of synthetic hairpins containing all possible combinations of dinucleotides following the recognition site and our results show that all sequences containing one adenine following GGATG were digested more efficiently. We further show that the same sequence preference is also observable in double stranded DNA at higher Mg^2+^ concentrations and even in optimal conditions. Kinetic results show that BtsCI has a noteworthy difference in the first-rate constants between different sequences and between the two catalytic domains. An increase in Mg^2+^ resulted in a drastic decrease in the catalytic activity of the top (sense) strand that wasn’t always accompanied by a nick in the bottom strand (antisense).

## Introduction

Restriction enzymes are elements of restriction-modification systems that protect bacteria and archaea from foreign DNA [1–4]. Since their discovery, they have become keystone tools in molecular biology and biotechnology [5,6]. Restriction enzymes are classified according to their subunit composition, cleavage site and its relative position to the recognition site, sequence specificity and cofactor requirements [7]. Types I and III are generally multimeric. Type I enzymes cut DNA at random distances to the recognition sequence and Type III enzymes recognize and cut separate non-palindromic sequences that are inversely oriented [8,9]. Type IV restriction enzymes recognize and cleave modified DNA [10]. On the contrary, Type II restriction enzymes produce discrete restriction fragments as a result of a very specific recognition and cleavage of DNA [11]. This characteristic makes them particularly useful for research applications. Despite their widespread use, the mechanism involved in the activity of most restriction enzymes is not completely understood. Magnesium ions are very often required as co-factors for catalysis but can be substituted, in some cases, by other divalent cations [7]. Despite being classified together, enzymes in each group are not necessarily related. Type II restriction enzymes do not always have similar amino-acid sequence, comparable three-dimensional structure or homologue functional domains. There are several subclasses of Type II restriction enzymes based on deviations from the general activity of the enzymes of this group. The Type IIS subgroup includes enzymes that cleave DNA outside their recognition site and constitutes one of the most diverse subclasses of endonucleases, with over 500 enzymes described so far (REBASE, http://rebase.neb.com/cgi-bin/asymmlist, 21 July 2017). Type IIS restriction enzymes may, like FokI, require to form homodimers to perform catalysis [12,13] but this is not the case for all restriction enzymes of this subtype, i.e. BtsCI and Mva1269I [14,15]. Instead, each monomer contains two independent cutting domains that act on the same substrate molecule.

The applications found for Type IIS restriction enzymes are varied, ranging from bacterial [16], viral [17] and human genome sequencing [18] to long oligonucleotide production [19–21] and production of overhangs for labelling [14]. A characteristic that some Type IIS restriction enzymes seem to share is a separation between the protein domains involved in substrate recognition and catalysis [13]. Commercially available Type IIS restriction enzymes are generally accompanied by information regarding the recognition site but are lacking in regard of the catalytic properties of the enzymes. In most of the reports and protocols involving enzymatic hydrolysis of DNA, restriction enzymes are expected to produce a defined restriction pattern. Nonetheless, unspecific cleavage is referred to in the literature as early as 1975 [22]. Sequence dependence has been implied for the cleavage of HphI [23] and Type IIS catalytic slippage has also been described for several enzymes [24]. REBASE provides a list of 37 Type IIS restriction enzymes for which variable cuts have been described, all being reported to hydrolyse DNA at different positions than what is canonically described (REBASE, http://rebase.neb.com/cgi-bin/varcutlist, 25 March 2018).

While working with a Monoclonal Stoichiometric (MOSIC) method to enzymatically produce oligonucleotides [19], we came across circumstances in which BtsCI and BseGI show an erratic activity when cutting hairpin structures, with incomplete cuts in at least one of the strands. Within the field of enzymatic oligonucleotide production the MOSIC approach is still the only possible solution if full design freedom is required for both 3’ prime and 5’ prime ends of each oligo as other methods like the one developed by Dietz et al.,[25] requires addition of DNAse domains at the oligo ends, which are partially released together with desired sequence. So while MOSIC can be done by adding a large amount of REases, an increased understanding of why some cuts are more difficult than others is desirable and could lead to cost reductions when it comes to high scale enzymatic oligonucleotide production. Here, we expand on the results following this observation and show that surprisingly, both BseGI and BtsCI have a sequence preference in the nucleotides flanking the recognition sequence when cutting DNA hairpin structures, but also when digesting double-stranded DNA.

## Materials and methods

### General remarks

Unless stated otherwise, all gels were imaged using an ImageQuant LAS 4000 (GE Healthcare) and all the gel analysis were performed using the QuantityOne (GE Healthcare) program.

### MOSIC method and library preparation

We prepared oligonucleotides using the monoclonal stoichiometric method (MOSIC) developed in our lab, according to a protocol described before [19]. Briefly, we incubated 20 μg of pPDGF plasmid DNA containing our insert (sequence in Data A in S1 File, ThermoFischer Scientific) with BsaI-HF (New England Biolabs(NEB)) for 4h in NEB4 buffer (50mM Potassium acetate, 20mM Tris-acetate, 10mM Mg-acetate, 1mM DTT). After gel electrophoresis in a 0.8% agarose gel in TBE 0.5X with incorporated EtBr, we gel extracted the band using GeneJet gel extraction kit (ThermoFischer Scientific). The extracted DNA was self-ligated using T4 DNA Ligase (ThermoFischer Scientific) in Rapid Ligase Buffer at 22°C for 20min followed by heat inactivation at 65°C for 10min. The reaction product was nicked with endonucleases Nt.BspQI and Nb.BsrDI (0.5 U/l, NEB) in 1× NEB3.1 buffer (100mM NaCl, 50mM Tris-HCl, 10mM MgCl_2_, 100μg/mL BSA) for 2 h at 50°C followed by 2h at 65°C and then we heat inactivated the enzymes incubating the reaction mixture at 80°C for 20min. We then amplified the resulting nicked circular DNA by using phi29 DNA polymerase (0.5 U/l, Fermentas) for 20h at 30°C in a 1× reaction buffer (33 mM Tris acetate, 66 mM potassium acetate, 10mM Magnesium acetate, 1% Tween 20, 10mM DTT) containing dNTP mix (1 mM each; Thermo Scientific) and increasing concentration of single stranded binding protein T4 gene 32 (100 ng/l, NEB). We digested the amplification products with BseGI. We performed the reactions at 55°C for 20h. We loaded the digested products and their corresponding undigested Rolling circle Amplification (RCA) products in Denaturing Polyacrylamide Gel (Acrylamide:Bis-Acrylamide 29:1 20%, Urea 8M, Formamide 20%, TBE 1X) and analysed them by electrophoresis at 180V for 1h. The gel was stained with SYBR Gold (immersion in bath with 1:10000 dilution in TBE1X for 15min). We acquired images by UV trans-illumination (UVITEC) and we analysed them with ImageJ.

We prepared the samples with required adapters to the 5’ and 3’ ends to make the DNA compatible with MiSeq Illumina system using a method previously described in the literature [26]. The sequencing data was trimmed using Trimmomatic, and analysed using a python script (see Data B in S1 File).

### BtsCI RM system cloning

The nucleotide sequences for BtsCI restriction enzyme (*btsCIR*) and the respective methyltransferase (*btsCIM*) were obtained from NCBI (Accession no GQ449683) and the plasmids were obtained from New England Biolabs (NEB). We decided to reclone *btsciR* into pBAD-Ara::cmyc-His (ampicilin^R^). We started by preparing the insert by PCR amplification of the *btsciR* sequence with the following oligos:

EF_NcoI_BtsCIR_Fw: 5´CCACCATGGGTAAACGAATTTTATACTTGCTAACT3´

EF_BglII_ BtsCIR_NO STOP_RV: 5´CCAAGATCTCAGAAAAACAGCGCTTCCAT3´ and a PCR program starting with 30s at 98°C followed by 30 cycles of 10s at 98°C, 30s at 68°C and 72°C for 45s, and finishing with 10min at 72°C. We used 0,2μL (0.4U) of Phusion High-Fidelity DNA polymerase (ThermoFischer Scientific) in 1μL of DNA template with 1μL of each of the aforementioned primers (final concentration of 0.5μM), 4μL of HF Buffer, 0.4μL of 10mM dNTPs and water up 20μL. It is important to notice that in the EF_BglII_ BtsCIR_NO STOP_RV oligo we do not include the STOP codon of the gene in order to obtain a fusion with a c-myc fragment and a His-tag, which were used for purification later. We digest the amplicon and pBAD-Ara::cmyc-His with FastDigest NcoI and FastDigest BgIII (both ThermoFischer Scientific) for 30min in Fast Digest buffer according to the supplier’s double digestion protocol. We treated the vector with alkaline phosphatase (NEB) at 37°C for 1h to avoid recircularization. A 1:5 vector:insert was incubated with T4 DNA Ligase (ThermoFischer Scientific) at 22°C O/N. The reaction product was dialysed to MiliQ water for 1h using dialysis disks.

The methyltransferase gene was obtained in the vector pACYC184. We electroporated pACYC184:btsciM (chloramphenicol^R^) into *Escherichia coli* D1210 (F^-^ mcrB mrr hsdS20(r_B_^-^ m_B_^-^) recA13 leuB6 ara-14 proA2 lacY1 galK2 xyl-5 mtl-1 rpsL20(Sm^R^) glnV44 λ^-^ lacI^q^ lacY^+^) and allowed the bacteria to grow for 1h in LB before plating in agar with chloramphenicol (25μg/mL). After confirmation of a successful transformation by Sanger sequencing, we electroporated the resulting bacteria (D1210+pACYC184::btsciM) with the pBAD-Ara::btsciR-cmyc-His-tag construct and repeated the growth procedure by plating in agar with chloramphenicol (25μg/mL) and ampicillin (100 μg/mL). Glucose was also added to the plates (0.2% final concentration) to avoid leakage from the pBAD-Ara promoter.

### BtsCI overexpression and purification

To produce the protein, we picked a single colony of the plate and used it to prepare 10mL of LB media containing chloramphenicol (25μg/mL) and ampicillin (100μg/mL) pre-inoculate that was incubated at 37°C O/N. We then inoculated 6 flasks of 500mL of fresh LB with chloramphenicol (25μg/mL) and ampicillin (100μg/mL) and incubated them at 37°C, following the bacterial growth until OD_600_ = 0.55. At this point we added 5mL of a fresh 10% (w/v) Arabinose to each flask to induce the protein production and incubated for 4h. We pelleted the bacteria for 10min at 5000 rcf and discarded the supernatant. The harvested cells were stored at -80°C before lysis by sonication, clarification by centrifugation and filtering prior to applying the sample to an ÄKTAxpress system for a two-step purification consisting of an IMAC affinity step utilizing the His-tag followed by a size exclusion chromatography step (SEC, gel filtration). The final step was generally performed in a buffer consisting of 20 mM HEPES, 300 mM NaCl, 10% glycerol and 0.5 mM TCEP at a pH of 7.5. The purity if of the purified protein was assessed by SDS- PAGE.

### Synthetic hairpin digestion

We resuspended all the oligonucleotides in deionized water. The following sequences were tested for BtsCI/BseGI digestion:

Long_oligo_hp: 5’ CGATATTGTTGTTTCAACCCATCCGCGCGAAGCGCGGATG**GG**AACCGTTTGTA TTGCTAGCATTTTA 3’

TT: 5’ TGTTTCAAAACATCCGCGCGAAGCGCGGATG**TT**AACCGTTTGTATT 3’

AT: 5’ TGTTTCAAATCATCCGCGCGAAGCGCGGATG**AT**AACCGTTTGTATT 3’

GT: 5’ TGTTTCAAACCATCCGCGCGAAGCGCGGATG**GT**AACCGTTTGTATT 3’

CT: 5’ TGTTTCAAAGCATCCGCGCGAAGCGCGGATG**CT**AACCGTTTGTATT 3’

AA: 5’ TGTTTCAATTCATCCGCGCGAAGCGCGGATG**AA**AACCGTTTGTATT 3’

TA: 5’ TGTTTCAATACATCCGCGCGAAGCGCGGATG**TA**AACCGTTTGTATT 3’

GA: 5’ TGTTTCAATCCATCCGCGCGAAGCGCGGATG**GA**AACCGTTTGTATT 3’

CA: 5’ TGTTTCAATGCATCCGCGCGAAGCGCGGATG**CA**AACCGTTTGTATT 3’

GG: 5’ TGTTTCAACCCATCCGCGCGAAGCGCGGATG**GG**AACCGTTTGTATT 3’

TG: 5’ TGTTTCAACACATCCGCGCGAAGCGCGGATG**TG**AACCGTTTGTATT 3’

AG: 5’ TGTTTCAACTCATCCGCGCGAAGCGCGGATG**AG**AACCGTTTGTATT 3’

CG: 5’ TGTTTCAACGCATCCGCGCGAAGCGCGGATG**CG**AACCGTTTGTATT 3’

CC: 5’ TGTTTCAAGGCATCCGCGCGAAGCGCGGATG**CC**AACCGTTTGTATT 3’

GC: 5’ TGTTTCAAGCCATCCGCGCGAAGCGCGGATG**GC**AACCGTTTGTATT 3’

TC: 5’ TGTTTCAAGACATCCGCGCGAAGCGCGGATG**TC**AACCGTTTGTATT 3’

AC: 5’ TGTTTCAAGTCATCCGCGCGAAGCGCGGATG**AC**AACCGTTTGTATT 3’

The hairpins were brought to 90°C for 10min and let to cool down to room temperature. We incubated the resulting DNA at 1μM with 150μM BtsCI or 0.25U/μL BseGI in Tris-acetate 33mM, K-acetate 66mM and Mg-acetate 10mM for 120min at 50°C or 55°C followed by heat-inactivation at 80°C for 20min. We loaded the resulting mixtures into denaturing polyacrylamide gels (20% Acrylamide:Bis-acrylamide (29:1), Urea 8M, Formamide 20%, TBE1X) and analysed the samples by electrophoresis, at 300V for 35min. The gel was stained with SYBR Gold (immersion in a bath with 1:10000 dilution in TBE1X for 15min). The analysis was performed by measuring the band intensities of the three strands in the reaction mixture using the QuantityOne (GE Healthcare) program. We used the band intensity to calculate product/substrate ratios and transformed the data into a heat map using matrix2png interface [27].

### Plasmid digestion

We digested 1 μg of pUC19 with 15nM of BtsCI or 0,125U/μL of BseGI at 50°C or 55°C in 33mM Tris-acetate and 66mM K-acetate with different concentrations of Mg-acetate for 2h followed by heat inactivation at 80°C for 20 min. We loaded the samples in a 2% agarose gel containing ethidium bromide (1 μg/ml; Sigma Aldrich) and analysed them by electrophoresis, at 70V for 120min.

To follow the reaction over time, we digested 1 μg of pUC19 with 15nM of BtsCI or 0,125U/μL at 50°C or 55°C in 33mM Tris-acetate, 66mM K-acetate and 20mM Mg-acetate for 2h and stopped the reaction by heat-inactivation (80°C for 20min) at different time points. We loaded the samples in a 2% agarose gel and analysed them by electrophoresis, at 70V for 120min.

### Kinetics of double-stranded DNA digestion by BtsCI

We resuspended the following oligonucleotides in deionized water:

TT_Top: 5’ CTTCGTCTGTGACTAACTGTGCCAAATCGGATG**AA**CTCACCAAATTATAGCCGTA 3’

TT_Bottom: 5’ AGCTACGGCTATAATTTGGTGAG**TT**CATCCGATTTGGCACAGTTAGTCACAGACG AAGCAGACCGAAATC 3’

GG_Top: 5’ CTTCGTCTGTGACTAACTGTGCCAAATCGGATG**GG**GTCACCAAATTATAGCCGTA 3’

GG_Bottom: 5’ AGCTACGGCTATAATTTGGTGAG**CC**CATCCGATTTGGCACAGTTAGTCACAGA CGAAGCAGACCGAAATC

Control_oligo: 5’ AAGCTCTCTAACCCTGATGACTCAACTCGCAAGGGTCGTTTT 3’

The activity of each cutting domain was tested separately. To do so, we mixed each nucleotide with its complement in 1:2 ratio where the limiting strand was to be analysed, i.e. to analyse the top strand, excess of the bottom strand was used. We also added a control ssDNA without recognition site to be used as reference in the normalization required for the gel analysis. The oligonucleotides were brought to 90°C for 10 min and let to cool down to room temperature. We incubated the resulting DNA for final concentrations of 0.2, 0.4, 0.8, 1.6, 3.2 and 6.4μM (of limiting strand) with 10 pM BtsCI in Tris-acetate 33mM and potassium acetate 66mM at pH7.9 for 3min at 50°C. We also pre-incubated a STOP solution (20mM EDTA, 2% glycerol, 82% Formamide, 0.02% Orange G) at 80°C for 3min. We then added the magnesium acetate to a final concentration of either 10mM or 20mM to the reaction mixture. The reaction mixture was then mixed with the pre-incubated STOP solution after 0, 30, 60 and 90s. For 0s we added the magnesium acetate to the STOP solution before the reaction mixture was added. We loaded the resulting mixtures into denaturing polyacrylamide gels (20% Acrylamide:Bis-acrylamide (29:1), Urea 8M, Formamide 20%, TBE1X), the gels were stained with SYBR Gold (immersion in a bath with 1:10000 dilution in TBE1X for 15min) and analysed the samples by electrophoresis, at 300V for 35min. We imaged the gels using an ImageQuant LAS 4000 (GE Healthcare). The analysis was performed by measuring the band intensities of the visible DNA strands in the reaction mixture using the QuantityOne (GE Healthcare) program. We measured the concentration of the limiting strand and followed its digestion, and thus, disappearance over time. We considered the concentration obtained for 0s to be the initial concentration. Using the control strand as normalization reference, we determined the concentration of product for each time point by subtracting the substrate concentration measured from the initial substrate concentration. By plotting the product concentration over time we can determine the initial rate of catalysis (V_0_) by determining the slope. We then plotted the v_0_ for each substrate concentration and fitted the Michelis-Menten equation [28] using the non-linear least squares method in Microsoft Excel and retrieved the maximum rate constant (V_max_) and the Michaelis-Menten Constant (K_M_). The first-order rate constant (K_cat_) was calculated by dividing V_max_ by the enzyme concentration.

## Results

### BtsCI and BseGI digestion of hairpins can result in incomplete digestion

**Fig 1:**
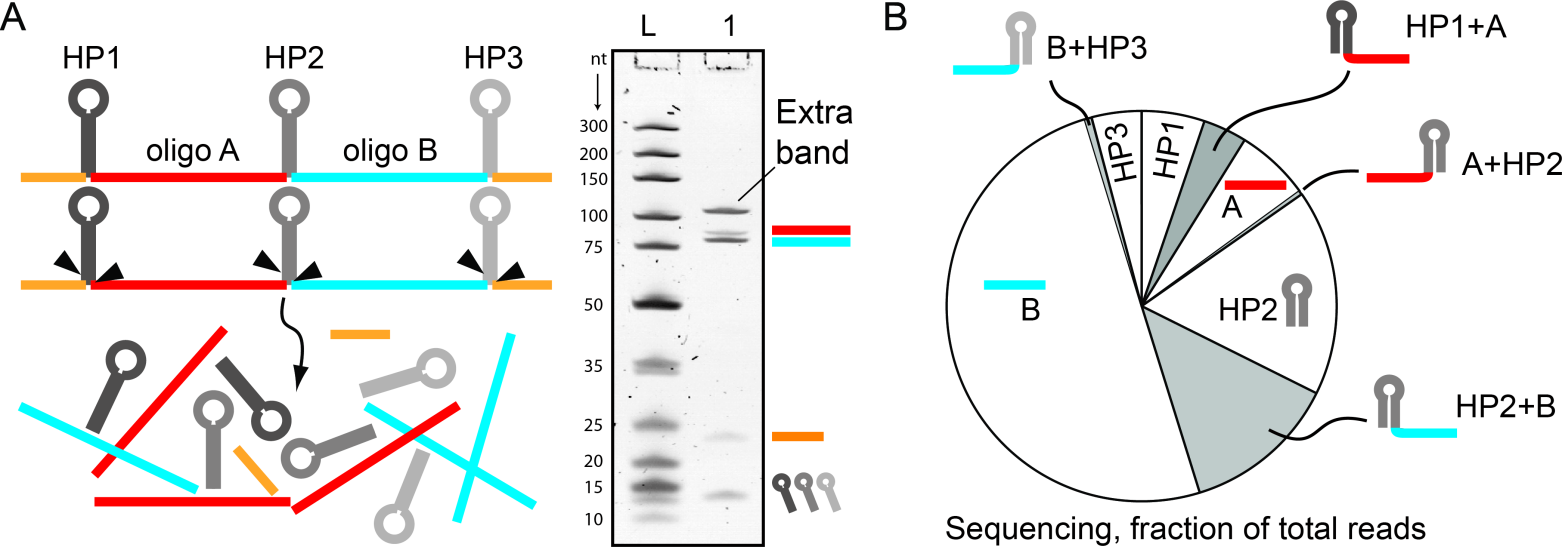
Incomplete cuts of DNA hairpins by BseGI. (**A**) Scheme of the digestion step of the MOSIC method (17) with a pseudogene containing two oligonucleotides of 80 (blue) and 86 nucleotides (red) intercalated by hairpins (black) with BseGI. The bands of 2 oligonucleotides, the hairpins and a MOSIC by-product (orange) bands are visible, together with an unexplained extra-band (Lane 1). The 10 bp-ladder was used for comparison (Lane L). (**B**) Next-generation sequencing results of the bands displayed in A, represented as fractions of the total reads. In the white sections are the expectable products of a full digestion. In the grey sections are the sequences that resulted from incomplete digestions.

In the process of producing high quality monoclonal oligonucleotides using the MOSIC method [19] we came across an unusual observation regarding BtsCI and BseGI digestion of hairpins. The MOSIC method is based on the amplification of a template using Rolling Circle Amplification (RCA) or phage production to obtain ssDNA. This template encodes for the oligonucleotide sequences intercalated with hairpin sequences that contain a recognition site of BtsCI and its isoschizomer BseGI. When amplified by RCA, the hairpin encoded in the ssDNA amplicons forms its secondary structure that will allow the restriction enzyme to digest the hairpins away, leaving only the single-stranded oligonucleotides. Although other secondary structures are likely to form, the hairpins are most likely by far the most stable component and it is difficult to see how additional erroneous cutting sites could ever be generated by spurious secondary structures. We designed a pseudogene containing 2 oligonucleotides of 80 and 86 nucleotides and intercalated them with hairpins (Fig 1A). After the amplification we digested the DNA with BseGI for 20h and analysed the digestion by denaturing polyacrylamide gel electrophoresis (PAGE). The digestion resulted in four bands, two bands of 80 and 86 nucleotides matching the oligonucleotides encoded in the template, one band of 23 nucleotides corresponding to the hairpins, another band corresponding to the by-product of the MOSIC method and a band of higher molecular weight (Fig 1A) that we could not identify. To analyse the reaction products better, we decided to sequence it using Next Generation Sequencing (NGS). We decided to use a pre-established method [26] to prepare a library of the digestion products with adapter sequences for NGS. The results showed the extra band was composed of a combination of the pre-designed oligonucleotides with one of the hairpins still attached.

**Fig 2:**
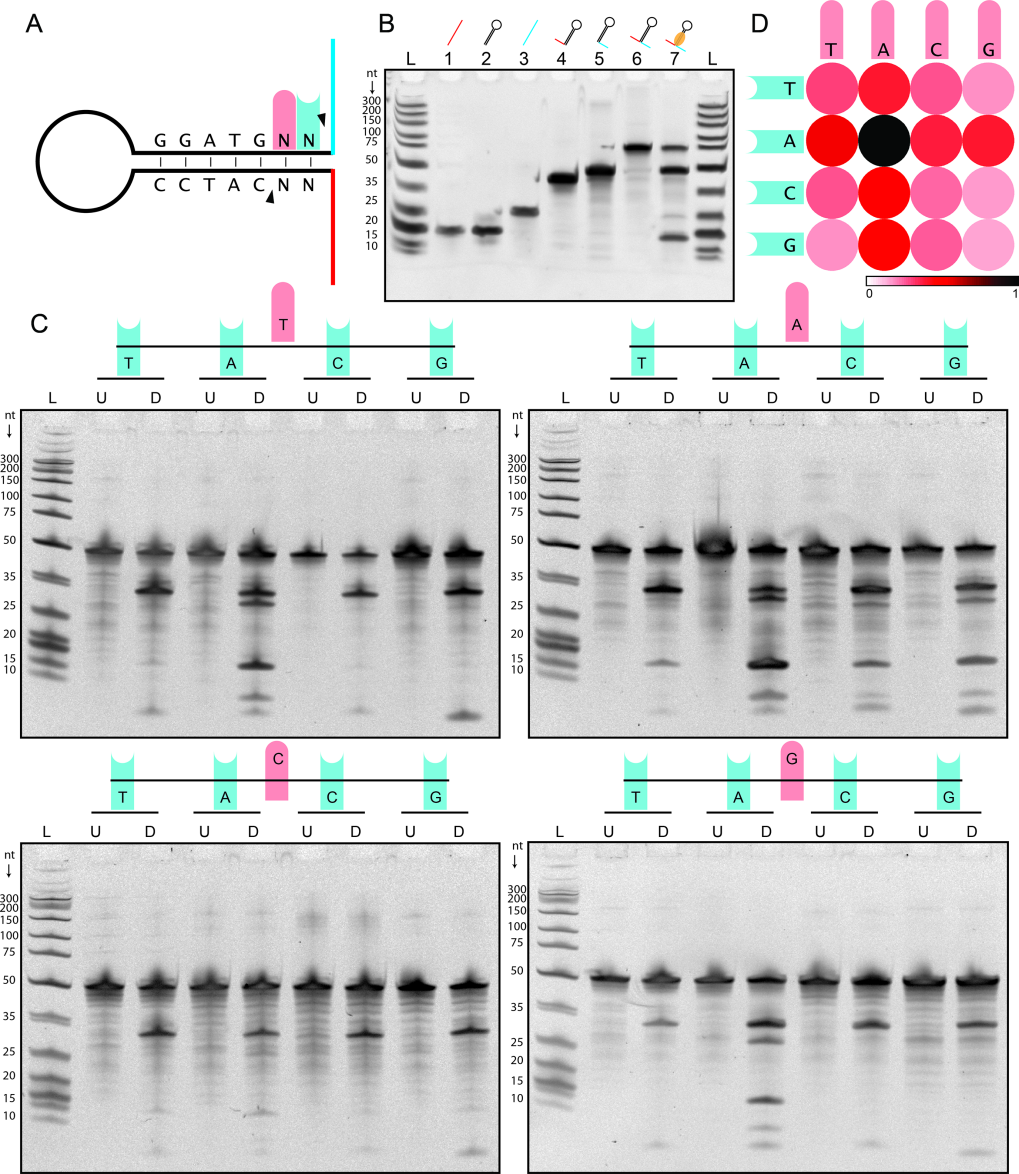
Synthetic hairpin digestion by BtsCI. (**A**) Schematic representation of the synthetic DNA structures digested with BtsCI. The hairpins used had oligonucleotides with different length attached to them for experiments showed in Fig 2B and 2C. Oligos A and B have 19 and 25 nucleotides, respectively, for experiment in Fig 2B and 10 and 13 nucleotides, respectively, in Fig 2C. The first base of the dinucleotide sequence is shadowed in red and the second base is shadowed in blue for future reference. (**B**) Digestion of a synthetic hairpin with BtsCI and the gel with digestion products. Together with the undigested and digested hairpins (Lanes 6 and 7), we loaded the red oligo, hairpin and the blue oligo (Lanes 1, 2 and 3) and the two potential incomplete cuts (Lanes 4 and 5). The Ultra-Low Range Ladder was used for comparison (Lane L). The dinucleotide sequence in the hairpin for this experiment is GG. (**C**) PAGE gels showing the products of the reaction of BtsCI in hairpins with different nucleotide combinations following the recognition site. The first base and second bases are shadowed in red and blue as in Fig 2A. U and D stand for undigested and digested products. (**D**) Colour code for the normalized product/substrate ratio after the reaction with BtsCI. The substrate (remaining undigested hairpin) and product (completely digested hairpins) bands from the gels in C were quantified and used to calculate a ratio to roughly estimate digestion efficiency. A darker colour represents a more efficient digestion and a lighter one represents a less efficient digestion. The red and blue shadows represent the same as in Fig 2A and 2C.

The main advantage of this approach was the possibility to identify oligonucleotides species in the digestion mixtures that, because of their molecular weight or low concentrations could not be resolved or visualized in the denaturing gel. Although the distribution of the sequenced digestion products can suffer some small inaccuracies due to putative library preparation bias associated with the method (such as variable accessibility of DNA oligonucleotides ends because of secondary structures), it is very clear how the most frequent incomplete cut is represented by oligonucleotides with a hairpin attached on its 5’ end (Fig 1B, full list of results in Data B in S1 File). This result is aligned with the PAGE results and in particular with the different intensities we found between the two bands of the fully digested 80 and 86 base long oligonucleotides. We wondered if BseGI’s isoschizoisomer, BtsCI, would provide similar results. To test this, we ordered a synthetic hairpin with the same sequence used in the MOSIC method with two oligonucleotides of 19 (red oligo) and 25 (blue oligo) nucleotides appended to it (Fig 2A). The recognition sequence on the hairpin was GGATGGG (oligo GG in the methods). After digestion for 2 hours with 150nM of BtsCI, we loaded the digestion product into a PAGE gel together with all the potential products of the digestion as controls (Fig 2B). From the gel it is clear that there is an incomplete digestion of the hairpin with a prevalent band corresponding to the hairpin with oligo B still attached to it in accordance with what we observed for BseGI in the NGS experiment.

### BtsCI and BseGI display sequence preference when digesting hairpins

To test our hypothesis, we ordered hairpins of all combinations of GGATGNN with two appended oligonucleotides of 13 and 8 nucleotides in single stranded form and incubated them for two hours with BtsCI or BseGI. After loading the digestion products in a denaturing polyacrylamide gel, we analysed them by electrophoresis (Fig 2C for BtsCI and Data C and Figure A in S1 File for BseGI). The gels showed distinct patterns for different sequences with a more complete digestion when NN contained at least one adenine next to the top band recognition site (C_t_). A notably better digestion is observed when AA was the dinucleotide combination in C_t_. For the remaining cases one band of higher molecular weight appears in the gel, as in Fig 2A. This data clearly shows that when digesting hairpins, there is difference between cutting the top strand and the bottom strand (C_b_) and that this difference changes depending on which dinucleotide sequence follows the recognition site. We analysed the gel bands to better understand which component was present in each digestion reaction by measuring the band intensity of product and substrate remaining. We compared the product/substrate ratio in each case. Looking at the graphical representation of the ratios (Fig 2D), an adenine dependence is evident. The best digestion is observed with AA but all other combinations containing one adenine in any position of C_t_ also show a better digestion when compared with the remaining sequences. We tested BseGI in the same set-up and found very similar results, with a clear preference for adenine being visible (Data C in S1 File). The results obtained with the synthetic hairpins seem to agree with the ones obtained with NGS. The hairpins used in MOSIC method experiment contained no adenine following the recognition site which, according to this data, would result in some of the least efficient reactions.

### BtsCI and BseGI show sequence preference when digesting plasmids in non-optimal conditions

**Fig 3:**
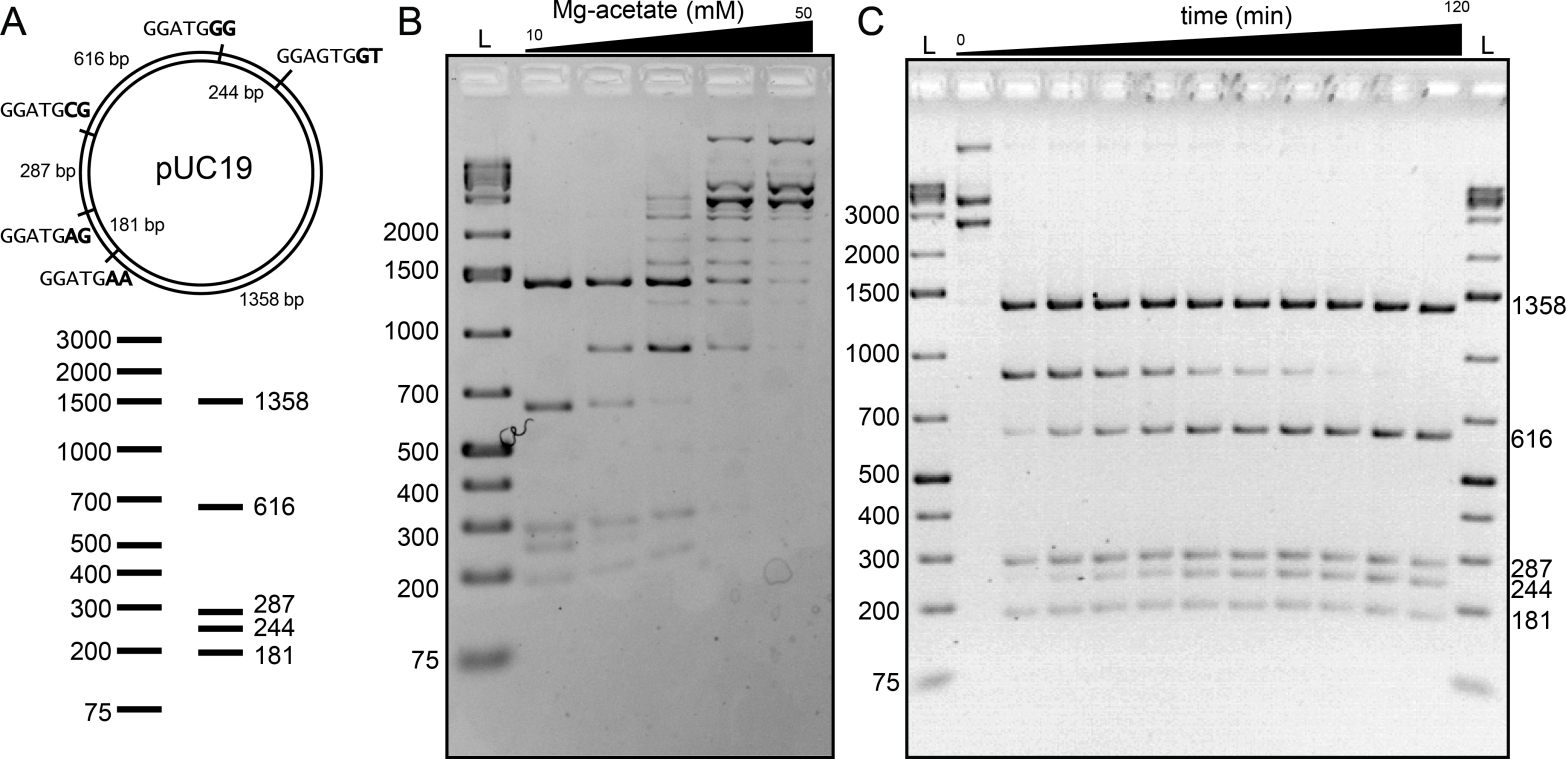
Double-stranded DNA digestion with BtsCI. (**A**) Scheme of pUC19 with 5 recognition sites for BtsCI/BseGI and the respective electronic digestion. (**B**) Effect of Mg^2+^ in the digestion of pUC19. The amount of Mg^2+^ in mM for each lane is represented. L represents the 1Kb plus ladder. (**C**) Time-lapse of the digestion of pUC19 with BtsCI in 20mM Mg^2+^.

Given the results we obtained with the digestion of hairpins, we questioned whether BtsCI and BseGI also displayed a similar sequence preference when digesting plasmid double stranded DNA. To assess this, we digested pUC19, a plasmid containing 5 recognition sites for BtsCI/BseGI, all of which are followed by a different dinucleotide pair (Fig 3A for BtsCI and Data D and Figure B in S1 File for BseGI). After a 2h digestion in optimal conditions (according to commercial suppliers) the plasmid was fully digested, in agreement to the available commercial information. We then speculated that the difference between the cutting of hairpin structures and plasmid could be approximated by pushing the conditions of dsDNA cutting to non-optimal conditions. We tested different conditions of salinity by performing the reactions in optimal conditions (10mM Mg-acetate) and 20, 30, 40 and 50mM Mg-acetate for 2 hours. The digestion products demonstrated an appearance of a faint extra-band at 30mM and a more pronounced band for higher Mg-acetate concentrations (Fig 3B). Due to its size, we determined it to be the result of the incomplete cut on the cleavage site containing the sequence CC at C_t_. Comparing this result with the results obtained when digesting hairpin structures, a clear similarity is evident because among the recognition sites present in pUC19, the one containing CC at C_t_ matches one of the sequences with least cleavage efficiency observed in the hairpins experiment.

In order to understand whether the incomplete cut of DNA by BtsCI was exclusively dependent on Mg^2+^ concentration or also a time dependent phenomenon, we performed a digestion of pUC19 in 20mM Mg-acetate and stopped the reactions at different time points up to 2h. We then measured the extent of the digestion by agarose gel electrophoresis. The resulting digestion (Fig 3C) shows the disappearance over time of the same band observed for higher Mg-acetate concentrations, and the concomitant appearance of the complete digested bands. After 5 min, the remaining bands are already fully visible and no other intermediate bands can be detected. This result suggests that a sequence preference of BtsCI and/or BseGI might occur regardless of the Mg^2+^ concentrations.

### Kinetics of BtsCI activity in dsDNA

The results on dsDNA prompted us to further the study of BtsCI activity in optimal conditions. To do so, we studied the kinetics of BtsCI. There is little to no information on BseGI available in literature and we did not receive any further clarification from the supplier, so we assumed it to be nearly identical to BtsCI. As for BtsCI, the data available is limited in terms of structure or function but the gene sequence and cloning procedure were available, which allowed us to produce the enzyme and, consequently, to have better control on the buffers and concentration of the enzyme employed in the kinetic experiments.

**Fig 4:**
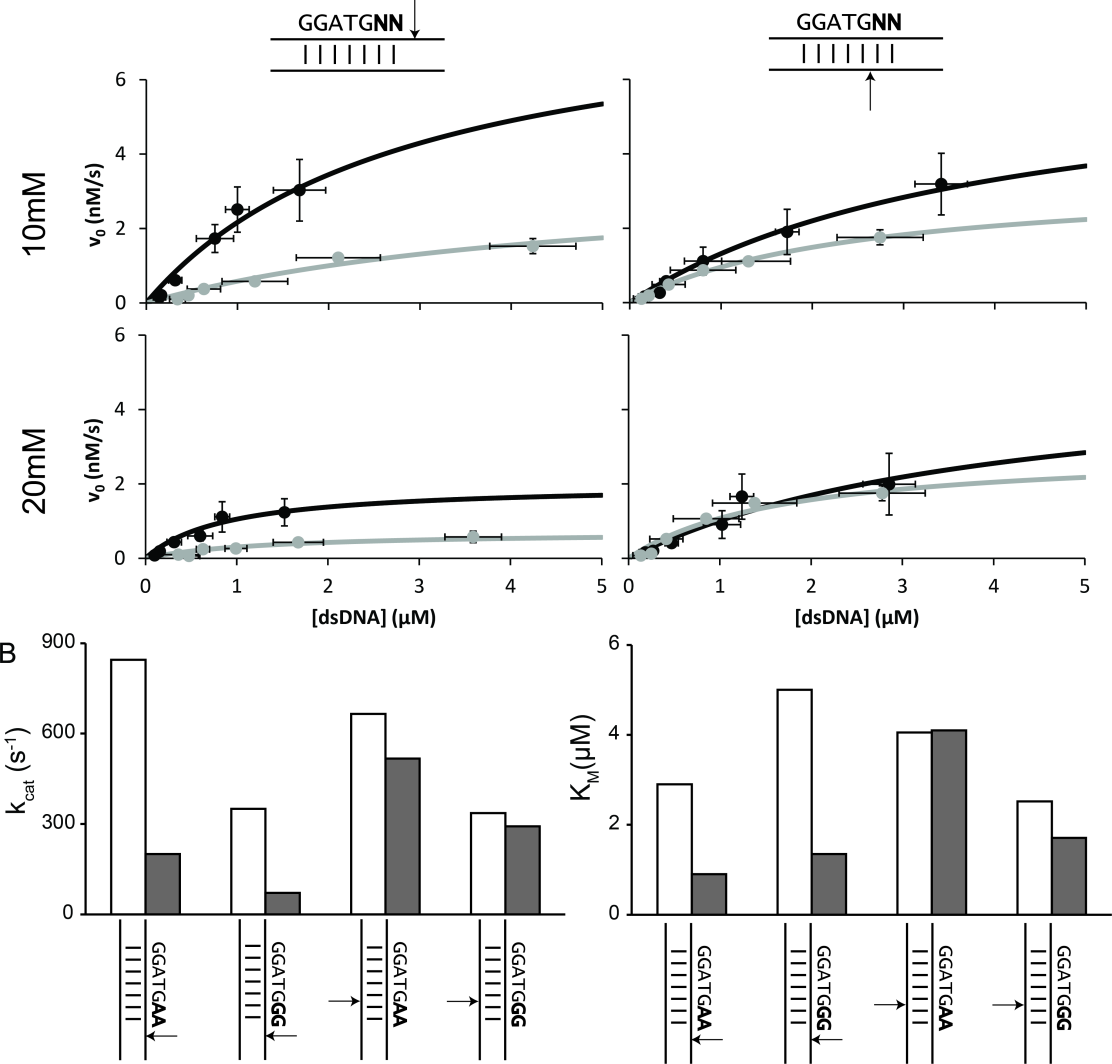
Kinetics of BtsCI in dsDNA. (**A**) Plots of the initial reaction rates (v_0_) for different substrate concentrations and the respective fitting to the Michaelis-Menten equation. The black dots represent the results for template with AA sequence and the grey dots represent the results for a template with GG sequence. Different rows represent different Mg^2+^ concentration and different columns show the results for either the top-nicking strand or the bottom-nicking strand. The error bars show the standard error from n = 3. (**B**) First order rate constant (k_cat_) and Michaelis-Menten (K_M_) constant determined from the curves in A. The white bars represent the reactions at 10mM Mg^2+^ and the grey bars represent the reactions at 20mM Mg^2+^ for each DNA substrate represented under the bars.

To comprehend the extent of the displayed sequence preference and to shed some light on the phenomenon observed in the hairpins and plasmid DNA, we studied the kinetics of BtsCI when facing a dsDNA substrate with the best observed cutting sequence (AA) and one of the worst (GG). We hybridized two synthetic strands of DNA. We analysed C_t_ and C_b_ in separate so as to independently assess the activity of both catalytic domains of BtsCI. This was achieved by having an excess of either of the strands in order to promote complete hybridization of the limiting strand that was being analysed. The hybridized strands also had different number of nucleotides to allow an independent analysis of the bands in the gel. We observed the disappearance of the substrate band in the first 90s of reaction at different substrate concentrations and used this information to calculate the amount of product formed (detailed explanation in Data E in S1 File). The product concentration was then plotted over time to allow for the determination of the initial rate of catalysis (V_0_). In turn, the plot of the different V_0_ determined for several substrate concentration can be fitted to the Michaelis-Menten equation (Fig 4A) [28]. With this data we were able to calculate the maximum rate of the reaction (V_max_) and the Michaelis constant (K_M_). We performed this at 10mM Mg-acetate and 20mM Mg-acetate to ascertain the effect of Mg^2+^ in the non-cutting observed in the plasmid DNA. We were able to calculate the first order rate (k_cat_) for each of the conditions tested by dividing V_max_ by BtsCI concentration (Fig 4B). The results show different k_cat_ values for the sequences tested. A higher k_cat_ was observable for the AA dinucleotide combination than for GG in both C_t_ and C_b_ at 10mM Mg-acetate. This result indicates that there is a differential digestion of dsDNA even in optimal conditions. Together with the data presented earlier in this work we can assert that the presence of the A in C_t_ (or T in C_b_) in one or both nucleotides following the recognition sequence results in the catalysis of DNA at a faster rate than with other nucleotide sequences. Furthermore, in both nicking sites, the reaction rates of C_t_ and C_b_ are comparable (C_t_ is slightly higher for AA and within the error for GG) suggesting that the cutting of both DNA strands occurs simultaneously, even if at different rates, for both sequence combinations tested. Additionally, when the Mg-acetate concentration is higher, the values of k_cat_ change differently according to sequence and nicking domain. The results show a very clear decrease of k_cat_ with increasing Mg^2+^ concentration for both C_t_ and C_b_. However, for C_t_ the decrease is more substantial than what is observed for C_b_ in both sequences. The addition of Mg^2+^ results in a 4 to 5-fold decrease for C_t_ and a 1.2-fold decrease for C_b_. The different extent of the reaction rate interference for both strands can be explained by the fact that BtsCI has two independent catalytic domains in the same polypeptidic chain [14]. Furthermore, this data demonstrates that increasing the amount of Mg^2+^ present in the reaction leads to a decrease in the reaction rate regardless of the sequence. It is worth mentioning that K_M_ also varies with the increase in Mg^2+^ concentration but in the opposite direction of k_cat_. According to the literature, a decrease in K_M_ is normal for higher salt concentrations, and it is related to increased binding efficiency between the enzyme and the template [24].

## Discussion

During this work we demonstrated that BtsCI and BseGI display a preference for adenine in the top strand of the dinucleotide sequence between the recognition site and the cleavage site. The NGS results on MOSIC extra bands together with the digestion of synthetic hairpins demonstrated that hairpin structures are digested differently according to the DNA sequence following the recognition site. In a less efficient digestion, C_t_ was shown to be uncut more often than C_b_. We also demonstrated that limited plasmid DNA digestion is verified at higher Mg^2+^ concentrations, with incomplete cuts occurring in agreement with the sequences observed for the hairpin sequence preference. A previous report characterized the phenomenon of slippage in Type IIS restriction enzymes, where a group of 14 restriction enzymes were shown to perform recurrent shifted cleavage of the target DNA when compared to the canonical cleavage site [24]. In the same study, the presence of adenine is reported to be more prevalent within the results obtained among some of the enzymes tested but not all. The slippage detected changed dramatically depending on which enzyme was tested which reflects the structural and functional variability of this group. In our NGS results for BseGI, we have also encountered a few cases where the DNA hydrolysis occurred in non-canonical sites but the frequency of these cases is negligible in comparison with the incomplete cuts observed when digesting hairpin structures.

Some Type IIS restriction enzymes have separate DNA recognition and cleavage domains [13]. We focused on the DNA cleavage by BtsCI, which contains two independent catalytic sites of different motifs: a SD-X_6_-E-X_14_-QR motif, responsible for the activity on the bottom strand of the substrate and is usually found in Type IIS endonucleases, and a PD-X_n_-EXK motif responsible for cutting the top strand of the target DNA and is associated to Type IIP restriction enzymes [11,14]. Having different domains in the same peptidic chain might explain why the activity of both motifs differs considerably at higher salt concentrations, in which case the incomplete cut we observed for BtsCI and BseGI might be shared by more monomeric restriction enzymes.

**Fig 5:**
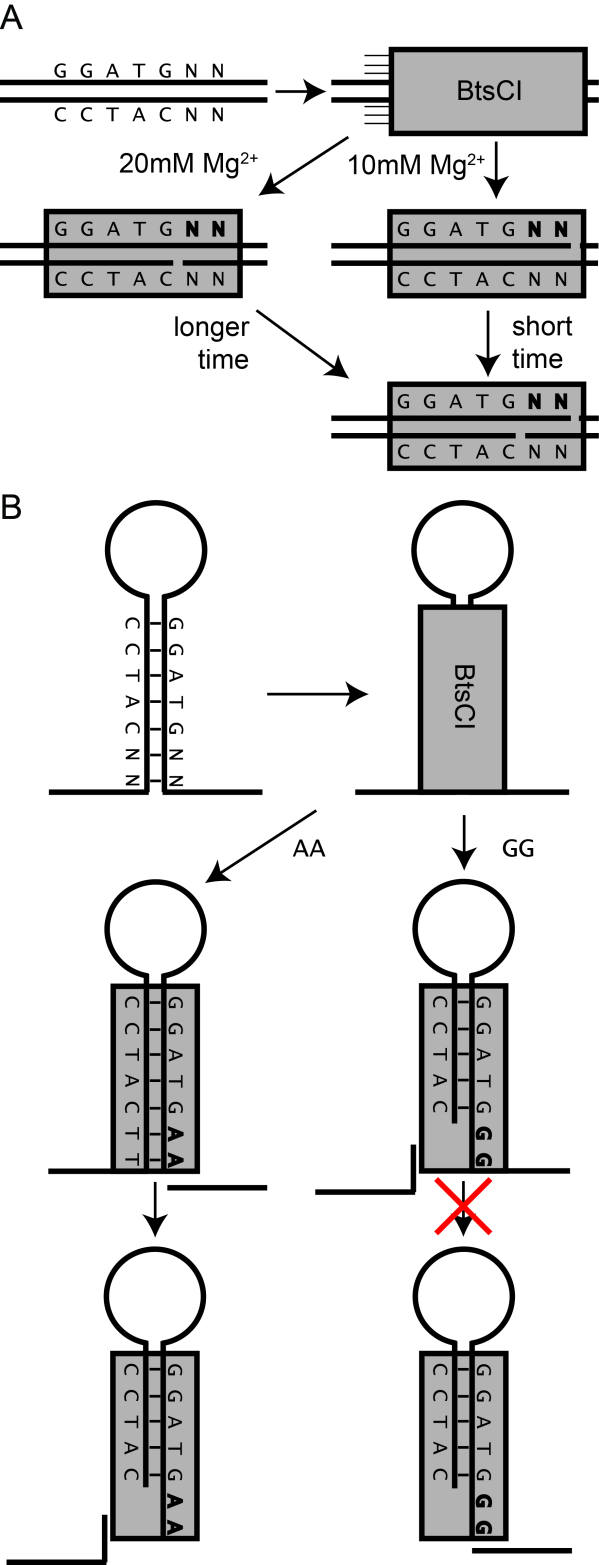
Scheme of the proposed mechanism for the incomplete digestion. (**A**) Different rates of cleavage at different salt concentration in dsDNA leads to a difference in cutting efficiency more predominant in the top-nicking strand, delaying the cutting. This interference is stronger in sequences that do not contain adenines. (**B**) In hairpin structures the sequence-dependence is more relevant because a change in cutting efficiency changes the order in which the nicks are made. This provokes the dismissal of the necessary double-stranded region and makes the second nicking invalid.

The kinetic studies we performed on BtsCI provide a clue about why the incomplete cuts may occur in a sequence dependent manner. At optimal conditions, BtsCI presents a higher reaction rate for both cleavage domains in the presence of an AA substrate than in the presence of a GG substrate, which proves that the enzyme cleaves some substrates better than others. Nonetheless, both domains of the enzyme cleave the substrate at comparable reaction rates, with C_t_ having a slightly higher activity for the AA template. However, when increasing the salt concentration from 10 to 20mM Mg^2+^, there is a 4-fold decrease in the activity of C_t_ and an almost negligible decrease on the activity of C_b_, resulting in a C_b_ activity at a much higher reaction rate than C_t_ for GG templates but still comparable for AA templates. This fact becomes more relevant if we consider the reaction occurs at 50°C for BtsCI (and 55°C for BseGI) (Fig 5A). In the case of the hairpins followed by ssDNA an early cut of the bottom strand results in having on oligo bound to the original structure by only 2 base pairs, reasonably insufficient to maintain the hybridization at that temperature. In the case of dsDNA, we can speculate that having a nick on the bottom strand can result in a change of conformation that makes it more difficult for C_t_ to perform the reaction (Fig 5B).

Another point to be made is related to K_M_. With an increase in Mg^2+^, K_M_ values drastically drops, which is normally associated to a more stable binding of the enzyme to the substrate. This suggests that even if the binding is better, the catalysis is still worse. We suggest that, regardless of whether the binding to the hairpin is similar for different sequences or not, the sequence preference observed is overwhelmingly dominant and it is the cause for the differential incomplete cuts observed.

## Supporting information

S1 File Supplementary information file. (DOC)
