# Supplementary material for: BtsCI and BseGI display sequence preference in the nucleotides flanking the recognition sequence
Source: PLoS One. 2018 Aug 17;13(8):e0202057. doi: 10.1371/journal.pone.0202057 (PMC6097692; doi:10.1371/journal.pone.0202057)
Supplement: S1 File — (DOC) [file pone.0202057.s001.doc]

**BtsCI and BseGI display sequence preference**

João Rosa^1^, Esther Fernandez-Gonzalez^1^, Cosimo Ducani^1^ and Björn Högberg^1,*^

^1^ Department of Medical Biochemistry and Biophysics, Karolinska Institutet, 17177 Stockholm, Sweden

* To whom correspondence should be addressed. Tel: +46852487036; Email:bjorn.hogberg@ki.se.

Supplementary information

A. Sequence of the insert used for the MOSIC method

GGTCTCACATTGCATAATTAACATCCGCGGAACGCGGATGTTTACTCAGGGCACTGCAAGCAATTGTGGTCCCAATGGGCTGAGTATGTGGTCTATGTCGTCGTTCGCTAGTAGTTCCTGGGCTGCACCATCCGCGGAACGCGGATGGTTCGAGGCGTAGAATTCCCCCGATGCGCGCTGTTCTTACTCAGGGCACTGCAAGCAATTGTGGTCCCAATGGGCTGAGTATCATCCGCGGAACGCGGATGATGCTCTTCATGCATTGGAGACC

The colour code for the sequence above is related to the colours used in Fig 1A of the manuscript. In orange are the sequences used during the MOSIC method that are by-products of the methods, in grey are the three hairpins used in this experiment and in red and blue are the oligonucleotides we wished to amplify.

B. NGS data analysis and results

To analyse the data obtained from the NGS facility, we ran the raw data through the following script:

import Bio

import re

import itertools

import csv

datafolder='Z:\\Joao\\NGS sequencing\\MosicSEQdata_reorganized\\'

fPDGF1='P976_1001_CTCTGCA_L001_R1_001.fastq'

fPDGF2='P976_1001_CTCTGCA_L001_R2_001.fastq'

# List of seqs [name,sequence, f_reads, r_reads]

tempseqs=[]

with open('PDGF.csv','r') as seqsFile:

csvr = csv.reader(seqsFile, delimiter=';')

for row in csvr:

tempseqs.append([row[0],row[1],0,0])

seqs=[]

for i in range(len(tempseqs)):

if i>0:

seqs.append([tempseqs[i-1][0]+'+'+tempseqs[i][0], tempseqs[i-1][1]+tempseqs[i][1], 0, 0])

seqs.append(tempseqs[i])

seqparse1=SeqIO.parse(open(datafolder+fPDGF1),'fastq')

seqparse2=SeqIO.parse(open(datafolder+fPDGF2),'fastq')

for s1, s2 in itertools.izip(seqparse1, seqparse2):

for item in seqs:

forw=len(re.findall(item[1], str(s1.seq)) )

rev=len(re.findall(item[1], str(s2.seq)) )

item[2]+=forw

item[3]+=rev

with open('output_PDGF.csv','w') as outFile:

csvw = csv.writer(outFile, delimiter=';', lineterminator='\n')

for item in seqs:

print item

csvw.writerow(item)

t=re.findall('CATCCGCGCTTCGCGCGGATGCTGGTAGTGCTATATTTCCTCTGTGCAACCATTCTTTTTTGTTT', str(s1.seq))

The results come in a csv format that can be open as a spreadsheet. The results obtained are represented in Table A-S1 file.

**Table A-S1 file. Total number of reads for each digestion product.**

| **DNA** | **Reads** | **% of total number of reads** |
| --- | --- | --- |
| *Hairpin 1* | 1064 | 5,2 |
| *Hairpin 1 + red* | 739 | 3,6 |
| *Red* | 1253 | 6,1 |
| *Red + Hairpin 2* | 64 | 0,3 |
| *Hairpin 2* | 3478 | 17,1 |
| *Hairpin 2 + blue* | 2615 | 12,8 |
| *Blue* | 10218 | 50,1 |
| *Blue + Hairpin 3* | 115 | 0,6 |
| *Hairpin 3* | 845 | 4,1 |
| *Total reads* | 20391 | 100 |

C. Hairpin digestions with BseGI: in these experiments the lanes are labelled with the reverse complement


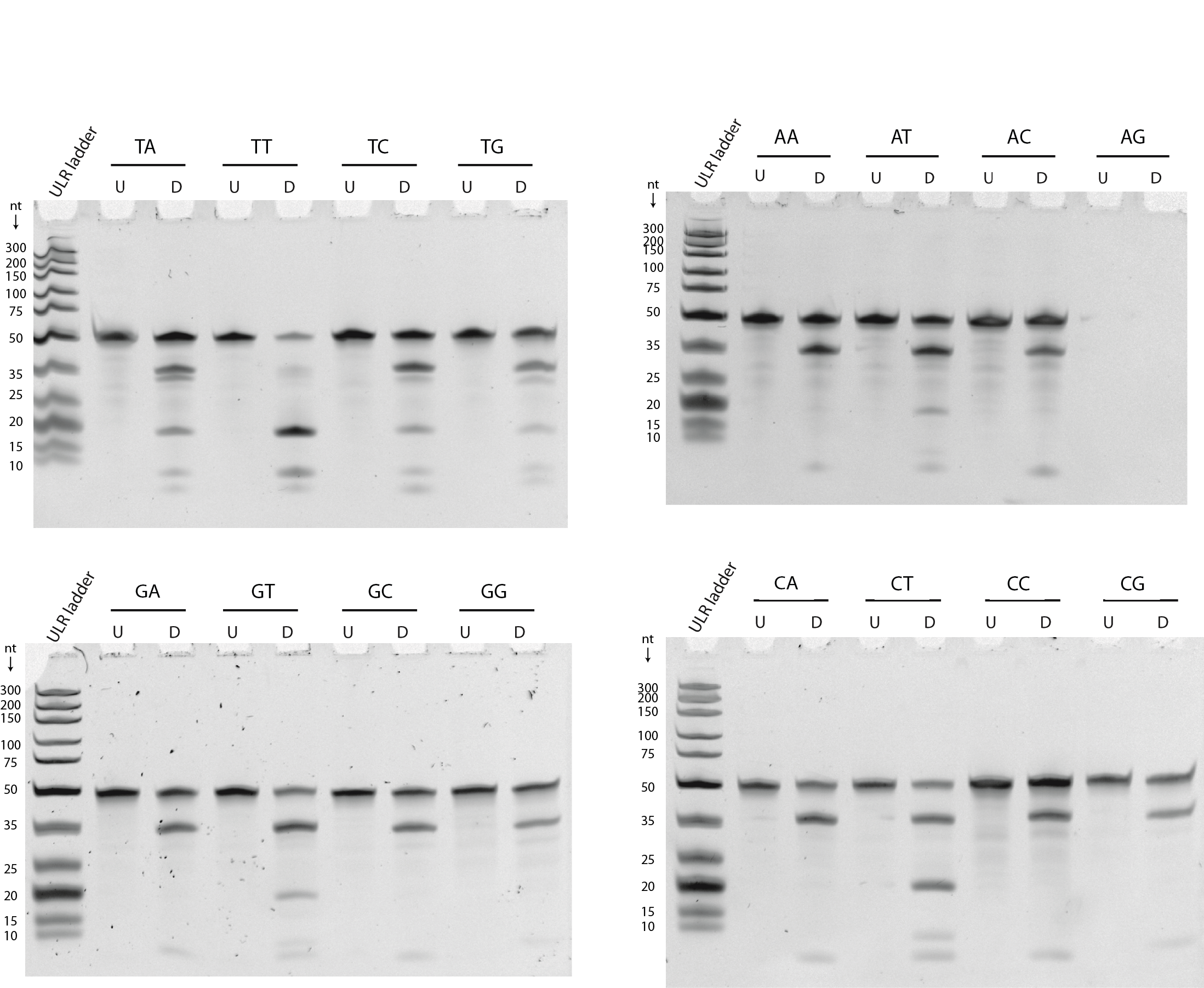


**Figure A-S1 file. Synthetic hairpin digestion by BseGI.** PAGE gels showing the products of the reaction of BtsCI in hairpins with different nucleotide combinations following the recognition site.

D – pUC18 digestion with BseGI


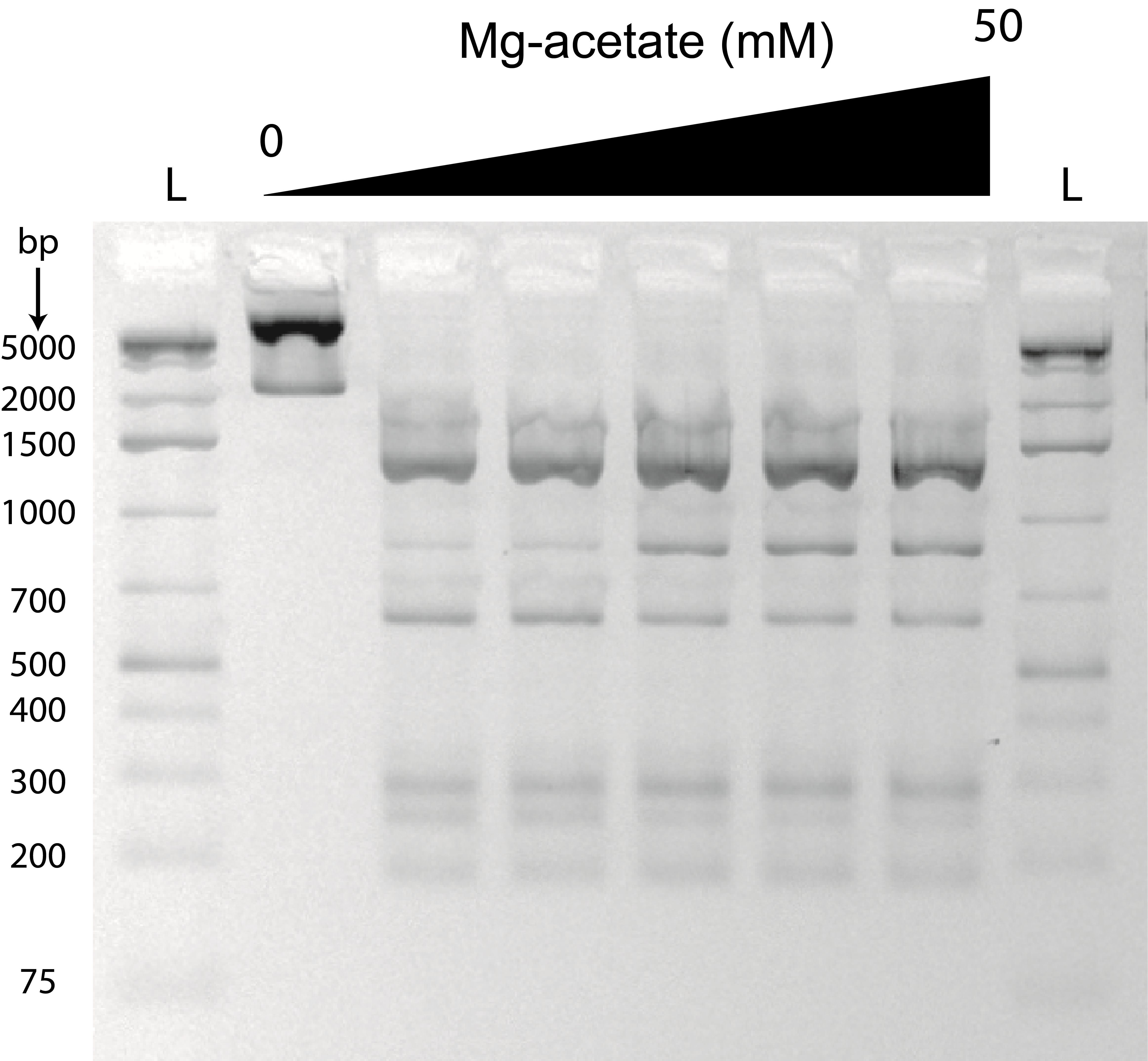


**Figure B-S1 file. Double-stranded DNA digestion with BseGI.** Effect of Mg^2+^ in the digestion of pUC19 by BseGI. The amount of Mg^2+^ in mM for each lane is represented. L represents the 1Kb plus ladder.

E – Calculation of v_0_

To calculate v_0_ for each substrate concentration of GG and AA at different salt concentrations, BtsCI was mixed with dsDNA synthetic template and the reaction was stopped at 0, 30, 60 and 90s as described in the materials and methods. The reaction products were loaded into denaturing PAGE as demonstrated below:


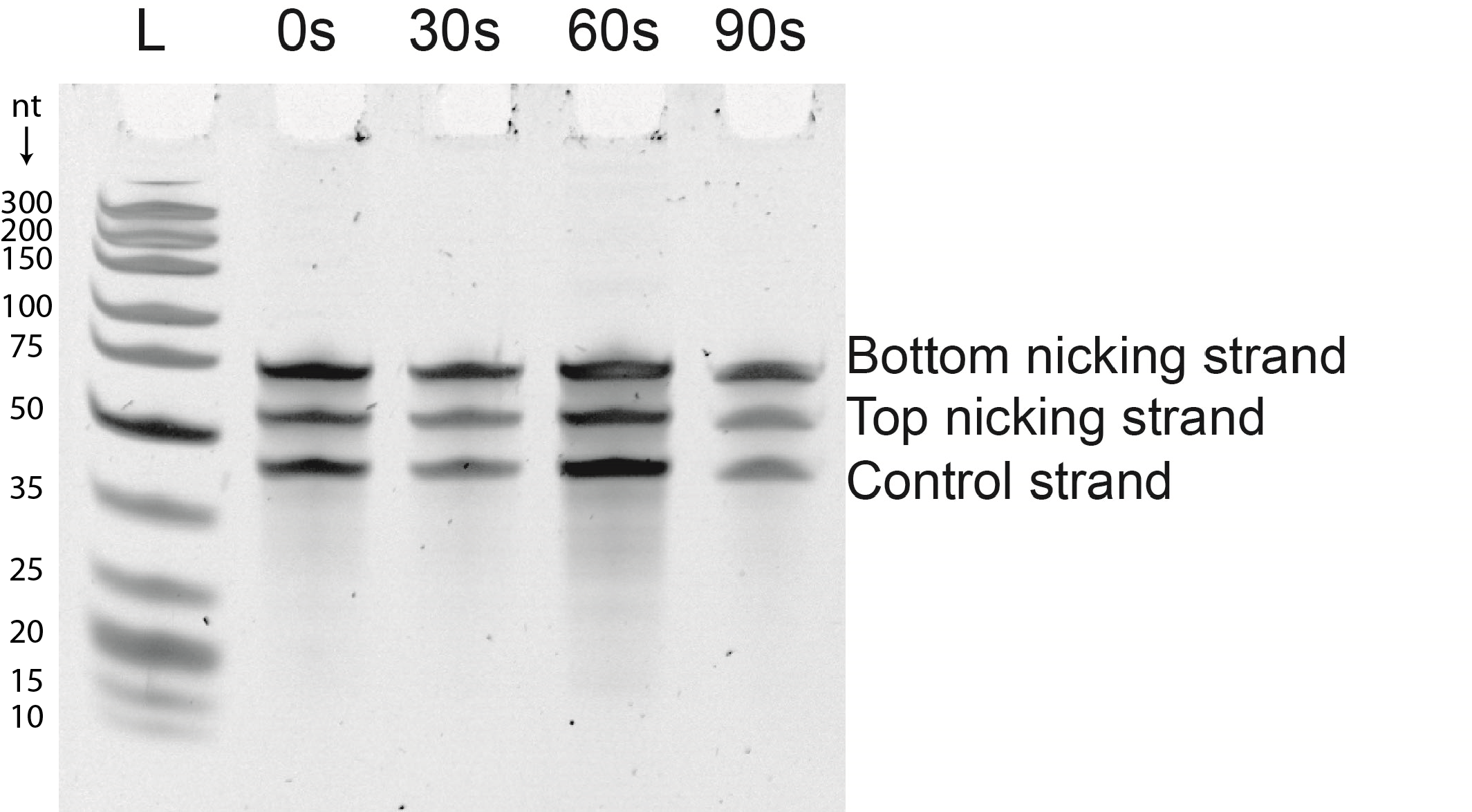


**Figure C-S1 file. Double-stranded DNA digestion with BtsCI over time.** Effect of Mg^2+^ in the digestion of synthetic dsDNA by BtsCI. The time at which the reactions were stopped is represented for each lane. L represents the ULR ladder.

To calculate v_0_, the initial reaction velocity, we have to measure the amount of product formed over time. Since the amount of product formed is too low to be detected by fluorescence, we observed the disappearance of the substrate instead. We measured the intensity of the nicking strand being tested and plotted it over time. By comparison with the ladder band we can estimate the concentration of each strand in each gel lane. To minimize the interference of the variability inherent to the pipetting of samples while loading them in the gel, we added a control strand that did not contain the recognition site of BtsCI, measured its intensity for each lane, and used that value to normalize the intensity of the bands being analysed.

Hence, the gel above translates to the graph below.


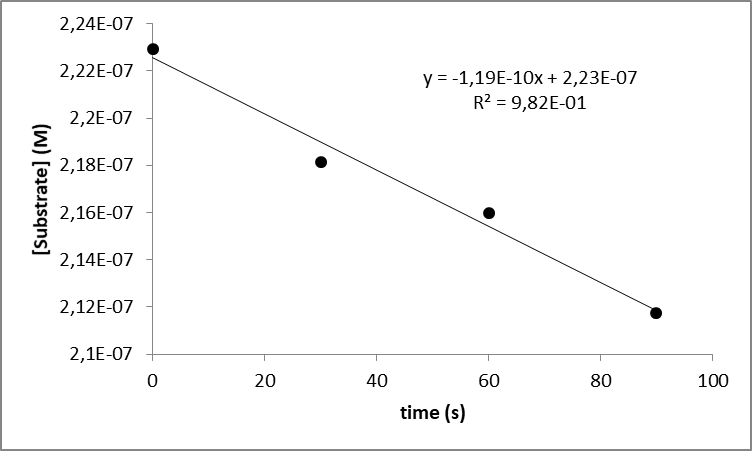


**Figure D-S1 file. Substrate concentration during the reaction with BtsCI.** dsDNA substrate concentration during digestion with BtsCI over time. The data is obtained from the intensity of the bands in Figure C-S1-file.

Finally, to determine the v_0_, we assumed the concentration of substrate measured for time 0s was the initial amount of DNA. We calculated the concentration of the product formed by subtracting the concentration calculated for each time point to the initial substrate. The final graph is displayed below. The slope of the curve represented by the time points corresponds to the v_0_ value.


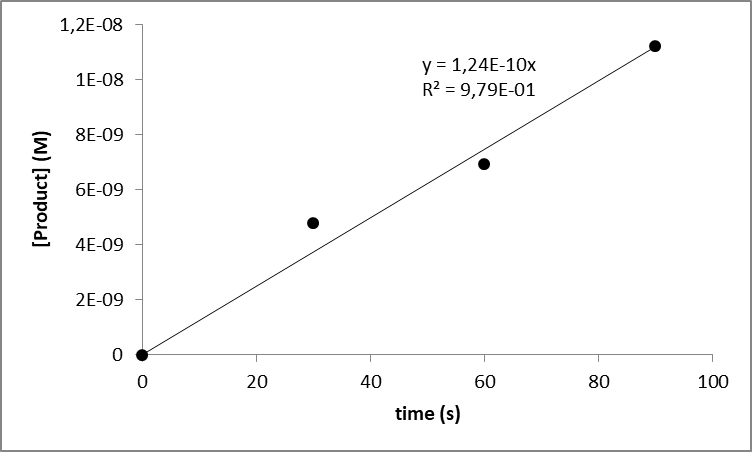


**Figure E-S1 file. Product concentration during the reaction with BtsCI.** ssDNA product concentration during digestion with BtsCI over time. The data is obtained using the initial concentration of substrate and the concentration determined in Figure D-S1 file.
